# Welding Spark Detection on Construction Sites Using Contour Detection with Automatic Parameter Tuning and Deep-Learning-Based Filters

**DOI:** 10.3390/s23156826

**Published:** 2023-07-31

**Authors:** Xi Jin, Changbum Ryan Ahn, Jinwoo Kim, Moonseo Park

**Affiliations:** 1 Department of Architecture and Architectural Engineering, Seoul National University, Seoul 08826, Republic of Korea; msnkimi2013@snu.ac.kr (X.J.); cbahn@snu.ac.kr (C.R.A.); 2 Department of Architectural Engineering, Gachon University, Seongnam-si 13120, Republic of Korea

**Keywords:** welding sparks, contour detection, deep learning, fire hazards, construction safety

## Abstract

One of the primary causes of fires at construction sites is welding sparks. Fire detection systems utilizing computer vision technology offer a unique opportunity to monitor fires in construction sites. However, little effort has been made to date in regard to real-time tracking of small sparks that can lead to major fires at construction sites. In this study, a novel method is proposed to detect welding sparks in real-time contour detection with deep learning parameter tuning. An automatic parameter tuning algorithm employing a convolutional neural network was developed to identify the optimum hue saturation value. Additional filtering methods regarding the non-welding zone and a contour area-based filter were also newly developed to enhance the accuracy of welding spark prediction. The method was evaluated using 230 welding spark images and 104 videos. The results obtained from the welding images indicate that the suggested model for detecting welding sparks achieves a precision of 74.45% and a recall of 63.50% when noise images, such as flashing and reflection light, were removed from the dataset. Furthermore, our findings demonstrate that the proposed model is effective in capturing the number of welding sparks in the video dataset, with a 95.2% accuracy in detecting the moment when the number of welding sparks reaches its peak. These results highlight the potential of automated welding spark detection to enhance fire surveillance at construction sites.

## 1. Introduction

Construction sites present numerous hazards that can lead to accidents, injuries, and even fatalities [1,2,3,4,5,6,7,8,9]. Welding-related activities, in particular, pose significant risks to workers, as welding sparks can cause bodily burns and fires at construction sites [10,11,12,13,14,15,16,17]. Specifically, welders are exposed to various dangers, including contact with overheated welding materials, intense light from arc welding, excessive ultraviolet and infrared radiation, and the emissions of molten sparks with molten metal [18,19]. Welding sparks themselves represent a significant fire hazard due to their intense heat and the presence of flammable materials. The high temperatures of these sparks can easily ignite substances such as fuels, solvents, gasses, oils, and combustible dust particles. Sparks can travel beyond the welding site, carried by air currents or wind, increasing the area at risk of fire. Accumulation of sparks in crevices or on combustible surfaces can further raise the chances of ignition, emphasizing the need for proper housekeeping practices. According to [20], it is necessary to monitor the welding process for more than 6 h, as welding sparks that remain bright and at high temperatures for over a minute pose a significant risk of causing major fire hazards. While most welding sparks extinguish spontaneously, those that persist can create potentially dangerous situations. Approximately 1650 fires per year, constituting 36 percent of all fires, are credited to welding torches based on statistical data. These incidents result in nine civilian deaths, which make up 42 percent of all fire-related fatalities [21]. The direct property damage caused by these fires amounts to approximately USD 157 million, representing 32 percent of the total property damage caused by fires each year in the United States [21]. Thus, construction safety plans to prevent fires in construction sites and injuries of construction workers represent an important research and safety management priority.

Recently, adopting modern technological solutions such as deep learning algorithms, computer vision, and sensor networks has created unprecedented opportunities to automate the process of identifying welding hazards beyond the inherent limitations (e.g., labor intensiveness and human errors) of traditional observatory approaches such as adding one more person to observe welding activities [22,23,24,25,26,27,28,29,30,31,32]. For example, Chen, W. et al. [33] proposed a progressive probabilistic transformer-based welding flame detection method. They compared five different features to detect welding flames and proved that their method can detect welding flames in real time. Separately, Zhen Zhong et al. [34] proposed a real-time convolutional neural network (CNN)-based flame detection model using closed-circuit television, achieving higher accuracy than traditional video-based fire detection and reducing false alarms. Similarly, Khan Muhammad et al. [35] presented a CNN-based flame detection method, outperforming conventional feature extraction methods and improving flame detection accuracy by 6%. Su, Y. et al. [36] proposed a CNN-based real-time construction fire detection system using video data. Their CNN-based real-time construction fire detection system for various construction environments (e.g., normal day, daytime fire, daytime welding, night normal, night fire, night welding, and night construction light) was proven superior to traditional smoke- and temperature-based fire alarms. Jadon, A. et al. [37] created a deep learning fire detection solution that does not require high-computational-capacity hardware, which is suitable for real-time applications and deployment on low-cost, moderate-performance devices.

Although the systems mentioned above include various fire scenarios in construction site environments, they do not include welding spark detection. Monitoring welding sparks is important to prevent a large-scale fire [38,39,40]. Detecting welding sparks using computer vision and deep learning, however, poses various technical and technological challenges [41]. The sparks are numerous, very small, and disappear quickly. Furthermore, detection of welding sparks is easily affected by brightness and shadow, in addition to color, shape, and motion [42,43,44,45,46,47,48,49,50,51,52]. In this context, we propose a novel method that incorporates color, shape, and motion-based feature engineering, while leveraging the power of CNN to select the best hue saturation value (HSV) color code automatically. This paper presents a significant contribution in the field of automatic welding spark detection by using contour detection with deep-learning-based parameter tuning. The proposed method addresses three important aspects: (1) automatic determination of optimized HSV threshold dictionaries using deep learning, (2) a reliable contour detection method to distinguish individual welding sparks, and (3) effective filtering of non-welding zones using deep learning and contour-area-based outlier removal to enhance detection accuracy. The experimental results and findings derived from this study offer valuable insights that can enhance the understanding and implementation of automatic welding hazard detection, ultimately contributing to the advancement of construction safety.

## 2. Methodology

Despite the existence of computer vision (contour detection) and deep learning algorithms to detect target objects in an image, their effective use for welding spark detection poses many challenges, as introduced in the Introduction. In this section, we describe an alternative method to detect welding sparks using contour detection methods and parameter tunings with deep learning models [53]. Contour detection methods can be effective for detecting objects with well-defined boundaries or distinctive shapes, such as welding sparks. By identifying and extracting the contours of sparks, it becomes possible to analyze their characteristics, track their motion, or perform further analysis and decision-making tasks [53]. The performance of this method can be critically affected by HSV codes among various variables, such as saturation max and value min values. In this regard, the proposed method aims to optimize a set of HSV thresholds using deep learning and various custom-made filters to better detect welding sparks. It includes (1) automatic HSV parameter tuning using deep learning, (2) distinguishment of welding sparks using contour detection, and (3) filtering of non-welding zones.

### 2.1. Automotive HSV Parameter Tuning Using Deep Learning

A You Only Look Once (YOLO) HSV threshold recommender is proposed to improve the process of finding the optimized HSV value. Although existing works [54] used a fixed HSV threshold dictionary to detect fire, the proposed welding spark detection model exploits an automotive HSV parameter tuning process. Since welding sparks are numerous and very small, disappear quickly, and are easily affected by brightness and shadow, the optimal HSV threshold dictionary for contour detection is different in each image. We first customized the YOLO algorithm. The YOLO algorithm employs CNN to detect objects in real time. The YOLO model is faster, with lower computational requirements, and more easily accessible when considering the real-time requirements of welding spark detection in our proposed system. Object detection in YOLO is conceived as a regression problem [55] and provides the class probabilities of the detected images [53,56]. The process of tuning the automotive HSV parameters consists of three steps: the HSV loop finder, YOLO welding spark detector in color masks, and confidence score sum-up. 

**Figure 1:**
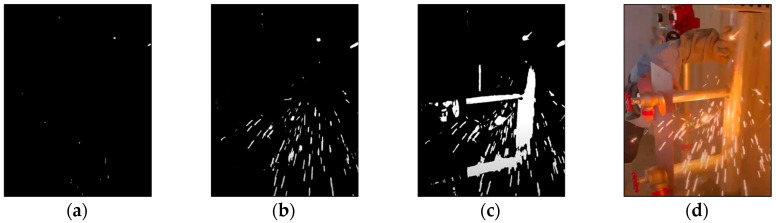
Examples of hue saturation value threshold loop finder of (**a**) loop 1, (**b**) loop 1000, (**c**) loop 2000, and (**d**) raw image.

Algorithm 1 shows the pseudocode of the HSV loop finder. The HSV loop finder uses saturation max and value min as nested loop indices, increasing from 5 to 255 with a 5 s interval. Other parameters for the HSV threshold dictionary are set as follows: hue min equals 0, hue max equals 179, saturation max equals 0, and value max equals 255. Video S1 demonstrates the loop process, showing the gradual decrease in the white area in the color mask. Figure 1a–c present the color mask images changed by running the HSV threshold loop finder. This generates 2601 color masks using the OpenCV library.
**Algorithm 1** Hue Saturation Value Threshold Dictionary Loop Finder1: **Input:** image = cv2.imread(“./welding01.jpg”)  2: **Initialize:** # Create ColorFinder object  3:             myColorFinder = ColorFinder(False)  4:             # Define lower and upper bounds for HSV loop finder  5:             hmin = 0  6:             hmax = 179  7:             smin = 0  8:             vmax = 255  9: **Repeat: for** s2 = (1, 2, 3, …, 51) **do**:  10:             **for** v1 = (1, 2, 3, …, 51) **do**:  11:               smax = s2 * 5  12:               vmin = v1 * 5  13:   list = [hmin, hmax, smin, smax, vmin, vmax]  14:   hsvVals = {‘hmin’: list[0], ‘hmax’: list[1], ‘smin’: list[2], ‘smax’: list[3], ‘vmin’: list[4], ‘vmax’: list[5]}  15:   # Update ColorFinder object  16:   imgColor, mask = myColorFinder.update(image, hsvVals)  17:   # Save color mask as image in folder  18:   cv2.imwrite(“./color_mask_output/{0}.jpg”.format(cnt), mask) 
**Output:** hsvVals(Hue Saturation Value Threshold Dictionary), color mask

**Figure 2:**
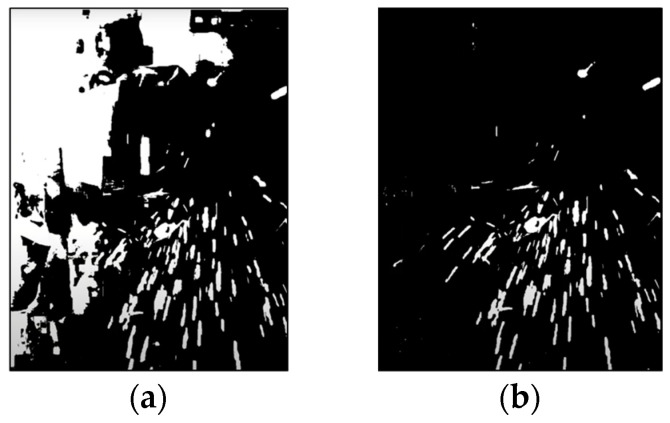
An example of white noise: (**a**) an image value of 100 and (**b**) an image value of 250.

This process incurs a significant computational cost of approximately 3 GByte per second. To mitigate the cost, we set the value min variable to 250 because we observe that there exist significant detection errors in the value min ranging from 5 to 245. Figure 2a,b illustrate example images with values set to 100 and 250, respectively. The disappearance of the white area trend can be observed at the top left of the image when the value min is 250. Notably, there is a significant increase in the white area when the value min surpasses 250. We collected 51 color masks with value min equals 250, which have the highest probability of containing the optimized color mask. 

The YOLO welding spark detector is trained and tested to find the best performed color mask image. It detects the welding sparks in the output color mask of the HSV loop finder. The labeling covers individual welding sparks in color masks for further selection of the optimized HSV color mask using sum-up confidence score value of each detected individual welding spark in each HSV color mask.The YOLO welding spark detector analyzes these 51 candidates color masks and provides a list of detected welding sparks, along with their confidence scores. The algorithm then calculates the sum of these confidence score values for each detected individual welding spark within each color mask. The optimized color mask is determined by selecting the color mask with the highest sum of confidence scores for detected welding sparks. On the other hand, the HSV dictionaries of the detected color mask are obtained using a self-incrementing variable in the HSV loop finder. Although manual tuning of HSV parameters for each image shows the best performance, it cannot be used in real-time welding spark detection in video, which is the main reason to propose the automotive HSV parameter tuning model. We confirmed that the automotive tuning process achieves 88% accuracy compared with ground truth of manual tuning of HSV parameters.

### 2.2. Distinguishing Welding Sparks Using Contour Detection

**Figure 3:**
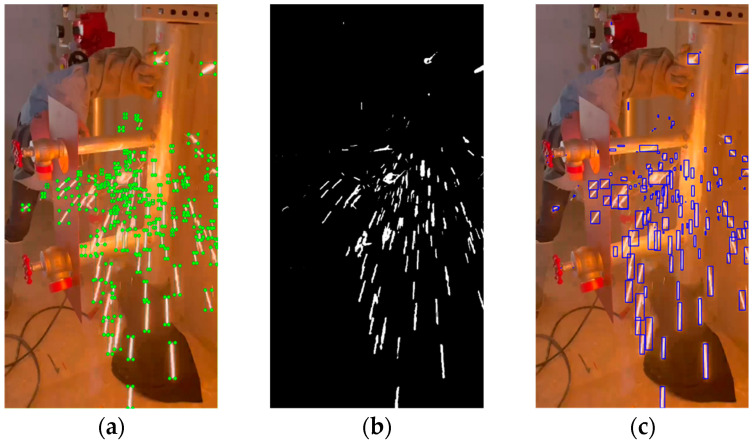
An example of welding spark detection using the contour detection method: (**a**) ground truth labeling sample; (**b**) hue saturation value color mask result; (**c**) welding spark detection result.

In the previous section, we selected an optimized HSV threshold dictionary in each image using deep learning. This dictionary enables us to determine the optimum color of welding sparks within each image. Subsequently, we gathered the pixels that satisfy the HSV threshold criteria from the image and connected all adjacent detected pixels to form a unified contour. Each contour was then converted into a rectangular bounding box, effectively representing the presence of a welding spark. Figure 3 shows an example of welding spark detection using the contour detection method.

Furthermore, we filtered welding sparks detected using predetermined thresholds as shown in Algorithm 2. Contour detection is designed to detect welding sparks using an HSV threshold dictionary. In the majority of situations, welding sparks are the brightest part in the corresponding RGB welding image, but the welding process also generates intense light that white surfaces can reflect. This intense light is the most common noise in the proposed welding spark detection color mask. If the welding is processed outdoors, sunlight is another noise source. Thus, we set two thresholds of the area of detected contour to eliminate noise in the proposed welding spark detection method: a number of pixels in bounding boxes below 1 and over 400 pixels. These thresholds were identified by sensitivity testing of the dataset.
**Algorithm 2** Contour Area Filter1: **Input:** image = cv2.imread(“./welding01.jpg”)  2:     hsvVals = {‘hmin’:0, ‘smin’:0, ‘vmin’:250, ‘hmax’:179, ‘smax’:135, ‘vmax’:255}  3: **Initialize:** imgColor, mask = myColorFinder.update(image, hsvVals)  4:         contours, hierarchy = cv2.findContours(mask, cv2.RETR_EXTER            NAL, cv2.CHAIN_APPROX_NONE)  5: **Repeat: for** cnt loop every contours **do**:  6:             area = cv2.contourArea(cnt)  7:             (x, y, w, h) = cv2.boundingRect(cnt)  8:             **if** area_threshold_1 < area < area_threshold_2:  9:               print(x, y, w, h) 
**Output:** (x, y, w, h) contour area

### 2.3. Filtering Non-Welding Zones

A welding zone filter based on a YOLO model is proposed to improve contour detection performance. The impact of light pollution, including reflections and intense light from welding, sunlight, and mirror reflections, was found to negatively affect the performance of the proposed model. Additionally, the diverse backgrounds found in construction sites posed challenges in accurately detecting individual welding sparks. For instance, the reflection of surrounding metals made it difficult to achieve precise welding spark detection, leading to potential inaccuracies. To mitigate this issue, a welding zone filter was developed to minimize the influence of varying backgrounds. This is the different YOLO model mentioned in Section 2.1. A welding zone is a rectangular area in a welding image containing the most welding sparks. The YOLO welding zone detector was trained using 300 RGB welding images, with 200 images used for training and 100 images used for testing. The welding zone was labeled using a labeling tool as the ground truth. The training parameters for the YOLO welding zone detector included 1 class, a batch size of 8, a learning rate of 0.04, and a total of 2500 training steps. The mean average precision (mAP) result of the YOLO welding zone detector is 72%. The output of the YOLO welding zone detector for welding zones includes bounding box coordinates in the form of x, y, w, h, along with a confidence score.

**Figure 4:**
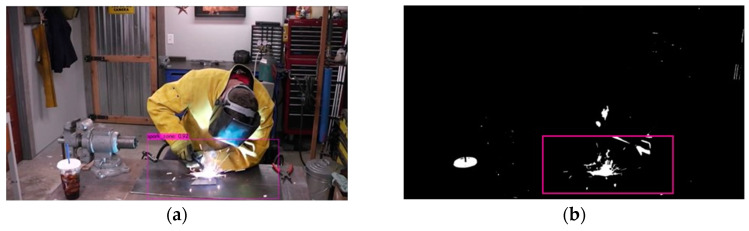
An example of welding zone detection: (**a**) welding zone in RGB; (**b**) welding zone in color mask.

Boundary coordinates of detected welding zones can be created as shown in Figure 4. For example, the proposed method detects a set of sparks and outputs their coordinates, yet the detection result frequently contains noises and error detection. Furthermore, the current deep learning method is not designed to work with two individual deep learning models. Thus, we stored welding zone detector output coordinates in x, y, w, h format, then sent them into the proposed welding spark detector. Then, the open computer vision library (OpenCV) draws a rectangle for display. We also used a set of if statements to filter out proposed welding spark detection coordinates that do not belong to the detected welding spark zone. Through this process, the proposed method can filter out the detection error of the proposed welding spark detection method.

The YOLO welding zone detector has the ability to identify the boundary coordinates of welding zones in each frame of a real-time welding video. By employing the optimized HSV threshold dictionary for contour detection in the welding video, it becomes possible to efficiently filter out erroneous detections from the YOLO welding zone detector, all in real time.

## 3. Results

### 3.1. Data Collection

A total of 100 welding videos were collected: 96 videos were from open-source platforms (YouTube), and 4 videos were from real-world construction sites. The videos were cut into 104 clips in total at the welding start point of the welding process, ensuring clear recognition of the beginning of the welding process. The mean and standard deviation of all welding video clips are 12.26 and 11.05 s, respectively. A total of 200 and 30 images were extracted from 104 videos for training and testing of the proposed approach, respectively.

### 3.2. Comparative Analysis of YOLO Models for Welding Spark Detection

In our real-time welding spark detection system, we chose to employ the YOLO model due to its advantages of speed, low computational requirements, and ease of access. To address various aspects of the detection task, we trained three custom YOLO models: a YOLO welding spark detector in color masks, a YOLO welding zone detector, and a benchmark YOLO welding spark detector. The training process for these custom YOLO detectors involved specific parameter settings. First, we assigned several classes as one, indicating the presence of welding sparks. Secondly, a batch size of eight was used during training. Thirdly, a learning rate of 0.04 was employed for optimization. Lastly, training was performed for a total of 2500 steps. For the YOLO welding spark detector in color masks, the model was configured to work with a single channel, since it focused on detecting welding sparks in color masks. On the other hand, the other two models utilized three channels, as they were designed to detect different aspects of welding sparks. All of these custom YOLO object detectors were trained using 200 images and evaluated on 100 images. Throughout the training process, transfer learning technology was employed to leverage pretrained models and enhance the detection performance.

As we mentioned, we used three customized YOLO object detectors for (1) the YOLO welding spark detector in color masks for automotive HSV parameter tuning, (2) the welding zone filter, and (3) the benchmark YOLO welding spark detector to compare with the proposed welding sparks model. We performed holdout validations of all YOLO models [57]. To train the YOLO welding spark detector in color masks, we utilized a dataset of 300 color masks. Specifically, 200 color masks (66% of the dataset) were used for training, while the remaining 100 color masks (33% of the dataset) were used for testing. The mean average precision achieved by this detector was 55%. The welding zone filter, which is another YOLO object detector, was trained using 200 welding images (66% of the dataset), and we evaluated its performance using 100 welding images (33% of the dataset). The welding zone filter exhibited a mean average precision of 72%. The benchmark YOLO welding spark detector was trained using 200 welding images and tested with 100 welding images, with an additional 30 welding images for evaluation. The precision obtained when tested with the initial 100 welding images was 12.28%. However, this precision improved to 48.85% when tested with the additional 30 welding images. Details of our training, testing, and validation methods can be found in previous fire detection systems that utilized image data, which are referenced in our work [41].

### 3.3. Welding Spark Detection in Images

In this study, a comprehensive evaluation was conducted to compare the effectiveness of the proposed HSV threshold contour detection method with a benchmark YOLO welding spark detector. Multiple-frame average precision (mfAP) was used as the evaluation metric. The mfAP reads all detected bounding box coordinates and calculates the intersection over union (IOU) and the precision.

The welding zone filter was designed to address the challenge of distinguishing between welding sparks and reflections from metal surfaces, which contour detection alone cannot achieve. By training YOLO to recognize the welding zone, we were able to eliminate incorrect detections in non-welding zones. The welding zone filter employs YOLO to identify the bounding box of the welding area. The rationale for the use of the YOLO model is that it is faster, with lower computational requirements, and easily accessible because it considers the real-time requirements of welding spark detection in our proposed system. By training YOLO to recognize the welding zone, we can effectively eliminate incorrect detections in non-welding zones. The proposed welding spark detection system achieved precision/recall of 27%/63.9% without filters (F1 score 37.9%). Adding a fixed contour area filter and welding zone filter increased precision to 44.5% (recall, 59%; F1 score, 50.7%). With a welding zone filter, a customized upper-threshold contour area filter for each welding image, and fixed lower-contour-area filter, the system yielded 74.85% precision and 56.6% recall (F1 score 64.5%). We evaluated the performance of these filters using a dataset of 100 welding images, with 30 images specifically chosen for this evaluation. These results demonstrate the effectiveness of the filters in improving the precision of the welding spark detection system.

Welding spark detection using contour detection with an optimized HSV dictionary including 30 welding RGB images achieved a precision of 74.45% and a recall of 63.5% (F1 score 68.5%). In contrast, the evaluation results for the benchmark YOLO model using the same set of 30 welding RGB images yielded a precision of 48.85% and a recall of 20.20% (F1 score 28.6%) when using a confidence score threshold of 0.7. These results indicate a significant improvement compared to the custom YOLO welding spark detection, with a 25.60% increase in precision and a 43.30% increase in recall.

### 3.4. Welding Spark Detection in Video

During real-time welding video testing, the YOLO HSV threshold recommender selects the most suitable color mask for each incoming frame. Subsequently, contour detection is conducted utilizing the optimized HSV threshold dictionary specific to each frame. Finally, the welding zone filter eliminates incorrect detections outside the welding zone for each frame.

To evaluate the effectiveness of the proposed welding spark detection method in real-time welding videos, we compared the number of detected welding sparks with the actual number of welding sparks in each frame. A total of 104 welding video clips were utilized to test the proposed welding spark detection method in real-time video scenarios.

**Figure 5:**
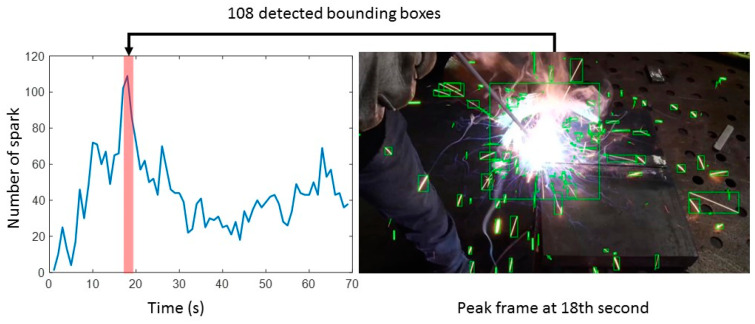
An example plot of the number of sparks in a video and an image of welding sparks at the peak point.

The proposed method processes each welding video clip and generates 104 sets of detected bounding box lists of welding sparks. The proposed method can generate a plot using the total number of detected welding sparks in one frame as the y value and the total number of frames in a welding video clip as the x value. Figure 5 provides an example of such a plot, where the x value of the plot also corresponds to the time in the video. Video S2 presents an example of real-time welding spark detection in a video.

Each peak in the plot represents a significant number of welding sparks detected by the proposed method. We can observe a large amount of sparks generated when the welding rod touches the steel. We performed peak detection tests on a total of 104 welding video clips to evaluate the efficacy of our proposed method for detecting welding sparks. The results revealed that the match rate of the detected frame number with the largest number of welding sparks and the real frame number with the largest number of welding sparks by the proposed welding spark detection algorithm was 95.2%. Among the five welding video clips in which the peak frame number of the plot (x value) did not match the frame number containing the largest number of welding sparks, further investigation was conducted. The reasons for failure in these five welding video clips include multiple welding activities within a single clip, changes in camera view during the welding video clip, and unsuccessful welding zone filtering.

## 4. Discussion

We proposed a novel method to automatically detect welding sparks using contour detection that combines color, shape, and motion-based feature engineering with the power of CNN-based parameter tuning. The key contribution of this paper is threefold. (1) Automatic determination of optimized HSV threshold dictionaries using deep learning: We leveraged the capabilities of CNNs to automatically select the best HSV color code for detecting welding sparks. This eliminates the need for manual tuning and improves the efficiency and accuracy of the detection process. (2) A reliable contour detection method for distinguishing individual welding sparks: We introduced a robust contour detection technique that effectively identifies and separates individual welding sparks from complex scenes. By leveraging color, shape, and motion-based feature engineering, we enhanced the accuracy of spark detection by up to 25.60% compared to the benchmark YOLO model. (3) Effective filtering of non-welding zones using deep learning and contour area-based outlier removal: We employed deep learning and contour area-based outlier removal techniques to filter out non-welding zones or false positives. This enhances the overall detection accuracy by reducing false detections and focusing on the relevant welding sparks. The results obtained from our study provide valuable insights into automatic welding hazard detection, thereby contributing to safety in the construction industry. By automating the detection process and improving accuracy, our method has the potential to enhance safety measures and reduce risks associated with welding operations.

**Figure 6:**
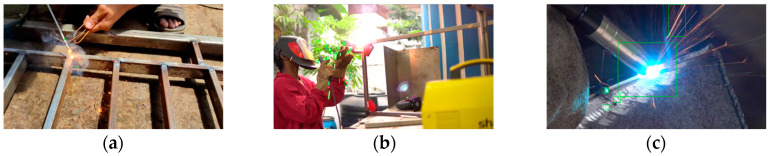
Example of images of (**a**) sunlight, (**b**) flashing light, and (**c**) reflection light.

This study has demonstrated the effectiveness of welding spark detection; however, there is still room to improve the accuracy of detection. While we have successfully determined the optimum HSV parameters using deep learning, there are other parameters, such as thresholds for contour area-based outlier removal, that can be further explored to increase accuracy. Additionally, we observed that the quality of the images can significantly influence the detection results. Images with higher performance levels exhibited clear welding sparks and appropriate environmental lighting conditions. In contrast, images with low detection accuracy displayed instances of welding sparks and various forms of light pollution, including flash, reflected light, low-quality videos, films behind sunglasses, and interference from sunlight, as illustrated in Figure 6. In future research, it is imperative to investigate the impact of light pollution present in the images on the detection results. Understanding how different types of light pollution affect the accuracy of the detection algorithm will provide valuable insights to enhance the robustness and reliability of welding spark detection in real-world scenarios.

We collected 100 welding images from 104 videos. A collection of 100 welding images was curated from various videos. This dataset contains diverse construction scenarios (e.g., outdoor welding, indoor welding, welding training, welding without proper personal protection equipment, robot welding, low-quality welding images, and filming behind sunglasses). While this dataset may appear to be too small to generalize, we argue that it should be considered as an acceptable size to test a new model. Previous works used 80 images to test indoor fire detection systems [41] and 40 images for smoke detection [41]. However, we also agree that the environments of construction sites are diverse and complex, so more image data with various environments from the real world should be further tested.

Automated fire surveillance systems may exhibit biases, both in terms of false positives and false negatives. Detection errors can be raised from image data quality. As we mentioned, we confirmed that light pollution (e.g., reflection, intense light of welding, sunlight, mirror reflection, etc.) diminished the performance of the proposed model. For example, surrounding metals are easily reflected and may trigger false classification. We developed two filters: a welding zone filter and a contour area filter with upper and lower thresholds. The welding zone filter was designed to address the challenge of distinguishing between welding sparks and reflections from metal surfaces, which contour detection alone cannot achieve. By training YOLO to recognize the welding zone, we were able to eliminate incorrect detections in non-welding zones. The contour area filter with upper and lower thresholds is a rule-based filtering process that quickly filters out incorrect detections based on specific threshold values. By combining these two processes, we enhanced the efficiency of filtering out erroneous detections. Future research is guaranteed to collect more diverse training data and develop additional filters (e.g., automotive upper threshold contour area filter) to increase accuracy.

Despite using a highly accurate and dependable model, misclassifications can still occur. In our future research, we aim to address this issue by developing a fire detection model that can be integrated with the welding spark model. This integration will serve to prevent fires that may arise from welding sparks. Video S2 showcases the initial fire detection model integrated with the welding spark detection model. In the video, it can be observed that the welding sparks eventually lead to a fire at 3 min and 35 s. Our model successfully detects both the fire and the welding sparks.

To enhance safety in high-risk construction environments, the proposed system for detecting welding sparks offers real-time monitoring of welding activities at the construction site. This can be achieved through the use of either closed-circuit television (CCTV) or a specially designed smartphone application. The welding video captured by the CCTV or smartphone app is then transmitted to a centralized database. Subsequently, the welding spark detection system retrieves the real-time welding video from the database and provides immediate feedback and alarms to both the construction site safety manager and the workers. This innovative approach enables the deployment of the welding spark detection system from a remote location, making it highly suitable and practical for hazardous construction environments. However, it is important to note that maintenance of the proposed welding spark detection system requires a good understanding of the Linux operating system.

Our proposed welding spark detection system relies on the utilization of OpenCV and YOLO. To use our system, users are required to first install OpenCV and YOLO. Once installed, they can proceed to copy our Python source code. For optimal performance, we recommend running the proposed welding spark detection on an Ubuntu Linux laptop with an Intel i5 6400 CPU, Nvidia GeForce 950M GPU, and 12 GB RAM or a higher-specification system. The processing time in Google Colab was optimized to be less than 0.5 s. Colab is a Jupyter Notebook environment running on a cloud virtual machine with a shared Nvidia Tesla A100 GPU. The proposed method showed the welding spark detection ability in a 25 fps video using the Ubuntu Linux laptop (Video S2). Additionally, the system requires a camera to monitor the welding process, which can be installed in a similar manner as setting up a webcam. The system can be powered using a standard electric power source or through the laptop’s battery.

For convenient monitoring and debugging of the proposed welding spark detection method, utilizing an integrated development environment such as PyCharm can prove highly beneficial. Furthermore, the proposed welding spark detection system can easily integrate with pre-existing fire alarm systems that rely on smoke sensors. This integrated setup enables dedicated monitoring of welding processes, while general fire safety monitoring can be efficiently managed by other computer-vision-based fire detection systems.

Although the primary focus of the proposed welding spark detection system is on detecting welding sparks, it is worth exploring the possibility of using the same method for detecting sparks generated during grinding and torch cutting. These processes may share similar visual characteristics with welding sparks. Further research is required to evaluate the feasibility of incorporating grinding and torch-cutting detection into the proposed method. When deploying the proposed welding spark detection system at construction sites, safety managers can efficiently monitor multiple welding processes simultaneously, thereby enhancing welding safety monitoring and overall efficiency.

Implementing the proactive detection of welding sparks using computer vision with deep learning can reduce the overall losses caused by fire hazards on construction sites. Additionally, the proposed welding spark detection system can aid construction safety managers in monitoring the welding process more effectively. According to general safety practice, a person is mandated to monitor the welding process and sparks. Once this method is fully implemented in actual construction sites, it can reduce the burden and error of the person who is monitoring the welding process.

On the other hand, some fire detection models have been implemented in actual industries, although the welding spark detection system has not been fully tested and demonstrated in actual construction because it is still in the exploratory phase of study. Prior to implementing the proposed welding spark detection model at a construction site, it may be necessary to integrate video privacy models, such as facial or body deidentification in images [58,59] to ensure compliance with privacy regulations. Additionally, it is important to consider potential issues such as detection errors and biased data. Furthermore, welding spark detection involves different challenging tasks compared to fire detection systems, for example, sparks are numerous, very small, disappear quickly, and are easily affected by brightness and shadow. Despite these challenges, to the best of our knowledge, this is the first study to demonstrate real-time welding spark detection (Video S2). We envision that our model can prevent casualties of fire disasters in construction sites once it is fully tested.

By leveraging computer vision techniques and deep learning algorithms, our proposed method enables automated monitoring of welding processes. It can detect and distinguish welding sparks in real time, providing early warning signs of potential fire hazards. We believe that by gaining recognition from construction regulatory bodies, the implementation of an automatic welding fire monitoring system based on our proposed method can significantly enhance the efficacy of fire hazard management. This system can act as a reliable and cost-effective solution to supplement or replace the need for dedicated personnel to monitor welding activities. It can help bridge the gap caused by limitations in budget and staffing, ensuring continuous and efficient monitoring of fire hazards during welding operations. This not only improves convenience but also minimizes potential risks and exposure to hazardous environments for personnel. By adopting our proposed method, construction companies can enhance their safety protocols, comply with regulations, and mitigate the risks associated with welding-induced fire hazards. The implementation of an automated welding fire monitoring system has the potential to revolutionize fire safety management in the construction industry, making it more efficient, cost-effective, and reliable.

## 5. Conclusions

In this research, a pioneering method is introduced to effectively detect welding sparks, which can potentially lead to catastrophic fire incidents at construction sites. The proposed approach includes the development of an automatic HSV threshold recommender system and the implementation of filtering methods, such as identification of non-welding zones.

The evaluation results demonstrate encouraging outcomes for the detection of welding sparks using the optimized HSV threshold dictionary. With an IOU of 50, precision and recall of 74.45% and 63.5% are achieved, respectively. These findings highlight the significant potential of automated detection systems for welding sparks in augmenting fire surveillance capabilities at construction sites.

Overall, this research contributes valuable insights, supporting the improvement of construction site safety by offering a reliable and efficient means of detecting welding sparks, thereby mitigating the risk of fire disasters.

## Data Availability

The data presented in this study are available upon request from the corresponding author.

## Supplementary Materials

Video S1: Hue saturation value threshold dictionary loop finder [https://youtu.be/ZHohedN-gxE] (accessed on 25 July 2023). Video S2: Welding spark detection [https://youtu.be/CY0Jh9E8ZCU] (accessed on 25 July 2023).
